# Individual Differences in Negative Emotion Differentiation Predict Resting-State Spontaneous Emotional Regulatory Processes

**DOI:** 10.3389/fpsyg.2020.576119

**Published:** 2020-11-10

**Authors:** Yali Wang, Chenyu Shangguan, Chuanhua Gu, Biying Hu

**Affiliations:** ^1^Department of Psychology, Zhejiang University of Finance and Economics, Hangzhou, China; ^2^Department of Psychology, School of Education, Shanghai Normal University, Shanghai, China; ^3^School of Psychology, Central China Normal University, Wuhan, China; ^4^School of Education, University of Macau, Taipa, Macau, China

**Keywords:** emotion differentiation, frontal alpha asymmetry, theta/beta ratio, spontaneous emotion regulation, resting-state

## Abstract

Negative emotion differentiation facilitates emotion regulation. However, whether individual differences in negative emotion differentiation is associated with resting-state spontaneous emotion regulation remains unclear. This study aimed to explore the effect of individual differences in negative emotion differentiation on spontaneous emotional regulatory processes as indexed by resting electroencephalogram (EEG) indicators (e.g., frontal alpha asymmetry and theta/beta ratio). Participants (*n* = 40, *M*_age_ = 21.74 years, 62% women) completed a negative emotion differentiation task. Afterward, 4 min of resting EEG data were recorded. Multiple regression results showed that negative emotion differentiation significantly predicted the alpha asymmetry at electrode pairs (F4–F3 and FP2–FP1) and the theta/beta ratio at the F3 and FZ electrode sites. Individuals with high negative emotion differentiation presented more left-lateralized activations and a lower theta/beta ratio. Taken together, these results suggest that individuals with high negative emotion differentiation show enhanced spontaneous emotional regulatory functioning. Thus, we provided the first resting-state neural evidence on emotion differentiation of spontaneous emotional regulatory functioning.

## Introduction

Generally, individuals can easily distinguish positive from negative emotions, but find it harder to distinguish between like-valenced emotions. The ability to distinguish between different like-valenced emotions is known as emotion differentiation (ED, also termed emotion granularity; Barrett et al., 2001; Kashdan et al., 2015). When identifying, labeling, or describing emotional experience, individuals with high ED tend to differentiate their emotions into fine-grained nuanced emotional categories. They report feeling angry in one situation and sad in another. In contrast, individuals with low ED tend to report their feelings in a coarse, less specific fashion, as simply “bad” or “good.” Specifically identifying what one is feeling provides information about emotional cause and context, which can facilitate emotion regulation.

Emotion regulation is the activation of a goal to up or down regulate either the magnitude or duration of the emotional response (Gross et al., 2011; Gross, 2015). Emotion regulation can be executed in controlled or spontaneous manners. Compared with controlled emotion regulation, spontaneous emotion regulation can be defined as the natural, unintentional, and relatively effortless process that results in changes in the properties (e.g., intensity, duration) of an emotional response (Jackson et al., 2003; Egloff et al., 2006; Gyurak et al., 2011). Spontaneous emotion regulation has been assessed by attenuated eyeblink startle magnitudes after the onset of unpleasant pictures (Jackson et al., 2003), continuous discomfort self-reports after negative emotional induction (Egloff et al., 2006; Ehring et al., 2010; Tortellafeliu et al., 2014; Eldesouky and English, 2019), and time needed to decrease heart rate or other physiological responses to baseline levels after emotion-inducing sessions (Hannesdottir et al., 2010). However, few studies have employed resting electroencephalogram (EEG) indicators to investigate spontaneous emotion regulation. Some specific resting EEG indicators, like frontal alpha asymmetry (FAA), and slow/fast wave ratios are closely connected to spontaneous emotion regulatory processing (Jackson et al., 2003; Goodman et al., 2013; Tortellafeliu et al., 2014). Thus, the present study aimed to examine the effect of individual differences in the ability of differentiating discrete emotional states on spontaneous emotional regulatory processes indicated by resting EEG indicators (FAA and slow/fast wave ratio).

### Emotion Differentiation and Spontaneous Emotional Regulatory Functioning

According to the perspective of feelings as information (Schwarz, 1990), emotions can provide a source of information on the environment. Individuals with high ED always experiences discrete emotions, which provide them with relevant information on the causes and consequences of emotions, such as the eliciting context and cognitive or physiological correlates (Barrett et al., 2001; Wranik et al., 2007; Ruys et al., 2012). Such critical emotional information might instruct them on how best to act in that emotional context spontaneously, thereby enabling them to spontaneously regulate their emotions in a more adaptive and effective way. The identification of discrete emotions could also assist in the selection of the most effective regulation strategies for those emotions. Thus, the ability to differentiate discrete emotions can facilitate spontaneous emotion regulation.

Moreover, a large body of empirical evidence has shown the spontaneous emotional regulatory function of negative ED (NED; the ability to precisely discern negatively valenced emotional states). High NED has been associated with the use of a wider range of emotion regulation strategies in situations of high-intensity negative emotions (Barrett et al., 2001). Meanwhile, lower NED has been more strongly associated with increased negative emotion compared with high NED (Erbas et al., 2014; Lennarz et al., 2018; Kalokerinos et al., 2019). Erbas et al. (2018) investigated which type of NED is most strongly related to the intensity of negative emotion, these categories included: between-category differentiation, the ability to make larger distinctions between very distinct emotions (e.g., anger and sadness); within-category differentiation, the ability to make fine-grained distinctions between emotions that are relatively closely related (e.g., anger and irritation); and integral differentiation, the ability to make distinctions between all emotions without taking into account their category membership. Their results showed that integral differentiation has the strongest negative relation to the intensity of negative affect and depressive emotion. Given their enhanced spontaneous emotional regulatory function of NED, individuals with high NED present a decreased negative emotion level. These findings indirectly proved that NED is associated with spontaneous emotion regulation.

Given its functioning of spontaneous emotion regulation, NED could reduce maladaptive self-regulation strategies, such as alcohol intake (Kashdan et al., 2010), attacks (Pond et al., 2012; Edwards and Wupperman, 2016), and non-suicidal self-injury (Zaki et al., 2013) brought by negative emotion. High NED may also reduce the impulsivity of individuals with borderline personality disorder (Tomko et al., 2015), weaken the association between rumination and depression (Liu D. Y. et al., 2019), and buffer the link between daily brooding and depression (Starr et al., 2017). Thus, theoretical and empirical evidence indicates that NED is associated with enhanced spontaneous emotional regulatory functioning. However, few studies have investigated evidence from resting-state EEG data.

### Frontal Alpha Asymmetry and Spontaneous Emotional Regulatory Functioning

Spontaneous brain oscillations in the resting state also remain very functionally active without external stimuli. Resting FAA reflects spontaneous emotional regulatory processes (Jackson et al., 2003; Goodman et al., 2013). FAA is usually calculated by subtracting the natural log of the left hemisphere alpha power from that of the right hemisphere; the power within alpha frequency bands (typically 8–13 Hz) is inversely related to actual cortical activations. Thus, higher FAA scores indicate more activations in the left hemisphere. Relative left frontal activity is associated with positive emotion approach and relative right frontal activity, with negative emotion withdrawal. Approaching positive emotions and withdrawing from negative ones are the outcomes of spontaneous emotion regulation; thus, the resting FAA is an indicator of spontaneous emotion regulation (Harmon-Jones and Gable, 2018). Moreover, many empirical studies have provided evidence on the relation between FAA and spontaneous emotional regulatory functioning. For example, Jackson et al. (2003) found that individuals with higher FAA (greater relative left frontal activity) presented greater attenuation of eyeblink startle responses after viewing negative stimuli, thereby providing preliminary evidence of resting FAA measures as an index of spontaneous emotion regulation. Goodman et al. (2013) reported that individuals with high FAA showed a higher spontaneous emotion regulation ability under high-stress situations. Wang et al. (2018) found that high FAA at the prefrontal cortex is associated with the frequent use of cognitive reappraisal. Resting FAA is also related to emotion regulation ability in children as reported by their parents (Hannesdottir et al., 2010) and cognitive reappraisal ability in anger-eliciting events (Perchtold et al., 2019). In addition, as FAA is associated with spontaneous emotional regulatory functioning, it is taken as a risk marker for emotion-related disorders, such as major depressive disorder (Stewart et al., 2011, 2014; Roh et al., 2020) and anxiety disorder (Adolph and Margraf, 2017). Altogether, FAA is suggested to underlie spontaneous emotional regulatory processes.

### Slow/Fast Wave Ratio and Spontaneous Emotional Regulatory Functioning

The resting EEG signal can be decomposed into the power of different frequency bands, including slow (e.g., δ: 1–3 Hz, θ: 4–8 Hz) and fast wave bands (e.g., β: 13–30 Hz). In addition to FAA, the slow/fast wave ratio is another indicator of spontaneous emotional regulation (Tortellafeliu et al., 2014; Zhang et al., 2018). For example, Tortellafeliu et al. (2014) demonstrated that individuals with a low delta/beta ratio showed high spontaneous emotion regulation ability.

The slow/fast wave ratio is indicative of spontaneous emotion regulation for the following reasons. First, the high slow/fast wave ratio (e.g., theta/beta ratio, TBR, which is calculated by dividing the theta wave power density by the beta wave power density) has been observed in children with attention deficit/hyperactivity disorder (Lansbergen et al., 2011; Arns et al., 2013). Individuals with high TBR demonstrate attenuated inhibition control of fear in emotional Go-No-Go tasks (Putman et al., 2010), report a lower level of attention control (Angelidis et al., 2016), and show a correlation with poor prefrontal cortex-mediated attentional and cognitive-emotional processes (Keune et al., 2017; Angelidis et al., 2018; Van Son et al., 2018). Thus, TBR is negatively related to emotional inhibitory control and attentional control function. Typically, slow wave oscillations have been linked to subcortical brain regions involved in affective processes, whereas fast wave activity is argued to reside at the cortical region and has been associated with cognitive control processes (Knyazev, 2007). A study using a decision task found that disadvantageous decision-making strategies were associated with higher TBR which suggests weaker top–down prefrontal regulation of subcortical drives (Schutter and Honk, 2005). Thus, slow/fast wave ratios may reflect the interaction of the frontal cortical and subcortical regions (Knyazev, 2007; Putman et al., 2010). Spontaneous emotional regulatory processing involves attention control and recruits the prefrontal cortex regulation of subcortically driven emotional responses (Ochsner and Gross, 2007; Morillas-Romero et al., 2015). The resting slow/fast wave ratio, therefore, can also be indicative of spontaneous emotional regulatory processing.

### Present Study

Despite the growing evidence of the association between NED and spontaneous emotion regulation, research has mainly focused on self-reported indicators of spontaneous emotion regulation. Few studies focused on the spontaneous emotion regulation indicative by the resting-state EEG oscillations. Self-reported indicators of spontaneous emotion regulation like adaptive use of emotion regulation strategies (Barrett et al., 2001), a reduction in maladaptive coping strategies (Kashdan et al., 2010; Pond et al., 2012), and a decrease in the intensity of negative emotions (Erbas et al., 2014; Lennarz et al., 2018; Kalokerinos et al., 2019) have been widely used in previous studies. However, it is generally believed that self-reported measurement indicators are easily affected by social desirability, expectation, and other factors, and are less accurate than objective physiological indicators to some degree. To this end, our study linked NED to the spontaneous emotion regulation indicative of physiological indicators. Given the previous evidence that some EEG-resting indicators (e.g., FAA and slow/fast wave ratio) index spontaneous emotion regulation (Jackson et al., 2003; Goodman et al., 2013; Tortellafeliu et al., 2014), the present study explored the relationship between NED and spontaneous emotion regulation of EEG-resting indicators to provide resting-state neural evidence for their relationship. Following previous evidence that NED can facilitate spontaneous emotion regulation, and findings that heightened FAA (Jackson et al., 2003) and decreased slow/fast wave ratio (Tortellafeliu et al., 2014) are associated with heightened spontaneous emotion regulatory functioning, we hypothesized that NED would positively predict resting FAA and negatively predict slow/fast wave ratio.

## Materials and Methods

### Participants

Forty-nine undergraduate and postgraduate students (18–26 years old) from Shanghai Normal University without any psychiatric, neurological, or medical illness were recruited as participants. Nine of them were excluded from further analysis for having excessive EEG artifacts (the number of removed epochs exceeded 20%). The final sample thus included 40 participants, including 25 women (21.84 ± 1.65 years old) and 15 men (21.57 ± 3.08 years old). Before data collection, all of the participants provided informed consent and the experimental procedures were approved by the Ethics Committee of Shanghai Normal University.

### Measures

We used a photo emotion differentiation task (Erbas et al., 2013) to measure NED. Referring to the work of Erbas et al. (2013), the experimental materials were 20 negative emotion pictures selected from the International Affective Picture System^1^ (IAPS; Lang et al., 2008). To ensure that these pictures were matched with 20 words to the maximum extent, we asked three participants to rate the matching degree using a five-point scale ranging from 1 (completely absent) to 5 (to a large extent). The average matching degree was 4.83 and average arousal level of these emotional pictures was 4.96. Twenty negative emotion words that matched the negative emotion pictures were used to measure NED: *fear, anxiety, anger, disgust, depression, sadness, loneliness, shame, frustration, hopelessness, panic, irritation, guilt, rage, dreariness, worry, unhappiness, nervousness, embarrassment, and regret*. We also used other self-reported measures of control variables, such as depression (measured by the Center for Epidemiological Survey Depression Scale; Radolff, 1977; Cronbach’s α = 0.80), state anxiety (measured by the State-Trait Anxiety Inventory; Spielberger et al., 1983; Cronbach’s α = 0.72), and handedness (measured by the Edinburgh Handedness Inventory; Oldfield, 1971).

### Procedures

**FIGURE 1:**
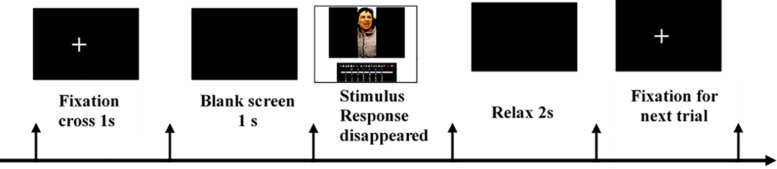
The trial structure for the emotion differentiation task. This is the scheme for each rating. During the photo emotion differentiation task, all 20 emotional words were presented one by one in a random order for each emotional picture. After the 20 emotional words were rated for one emotional picture, the next emotional picture was presented. This task included 20 emotional pictures in all and each picture needed 20 emotional word ratings, thus it included 400 trials in total.

The trial structure for the photo emotion differentiation task is illustrated in Figure 1. The task included 20 emotional pictures and each picture included 20 emotional words, thus the task included 400 trials in total. For each emotional picture, all the 20 emotional words were presented one by one in a random order. All stimuli were presented on a color monitor using E-prime 2.0 (Psychological Software Tools, Pittsburgh, PA, United States). The participants were seated in front of a monitor at a distance of approximately 50 cm from the screen. In the task, each trial began with a 1-s fixation cross. Following this, a blank screen was presented for 1 s followed by the stimuli. The stimuli were composed of an emotional picture at the top and an emotional word at the bottom. When the stimuli were presented, the participants were asked to rate the extent to which they experienced each emotion category in response to each picture using a seven-point scale ranging from 1 (completely absent) to 7 (to a large extent). After the 20 emotional words were rated against one emotional picture, the next emotional picture would be displayed. The presentation order of the pictures was also random. The stimuli disappeared after their response. At the end of each trial, the participants had 2s to rest. To ensure the participants to rate carefully, an attention check was performed by presenting three emotion words and, asking the participants to select which one appeared in the previous Photo Emotion Differentiation task at the end of the task.

To protect the resting EEG activities from any effect of the NED task, we first seated the participants in a sound-attenuated laboratory to record resting EEG activity. Four minutes of resting EEG data were recorded; the participants had their eyes closed for 2 min and eyes open for 2 min. The order of eyes-closed vs. eyes-open conditions was counterbalanced across participants. The instructions for the resting EEG recordings were as follows: “In the following experiments, you need to keep calm with eyes closed or eyes open. The order of the eyes-open and eyes-closed states is alternate. At the end of 1-min eyes-closed recording, you will hear the sound of ‘di’ to remind you to open your eyes. At the end of 1-min eyes-open recording, you will see the instruction presented in the screen to remind you to close your eyes. Please try not to think of any other things or move your head during the recordings.” Following the resting EEG recordings, the participants were asked to complete the NED task to confirm their level of NED. Lastly, some self-report measures as control variables, such as depression, anxiety, and handedness were reported. Other tasks (e.g., emotion-regulation tasks) were also conducted after the resting EEG data acquisition.

### Electroencephalogram Recording and Quantification

EEG data were recorded using 64 Ag–AgC1 scalp electrodes placed according to the international 10–20 system (NeuroScan 4.3, Inc., United States). The reference electrode was placed on the left mastoid, and the ground electrode was located between the FPZ and FZ. Electrodes placed above (one electrode) and below (one electrode) the right eye recorded the vertical electrooculogram and electrodes located on the outer edge of either eye recorded the horizontal electrooculogram. Signals were amplified using a 0–100 Hz band-pass filter and continuously sampled at 500 Hz/channel. Electrode impedance was maintained at below 5 kΩ.

**FIGURE 2:**
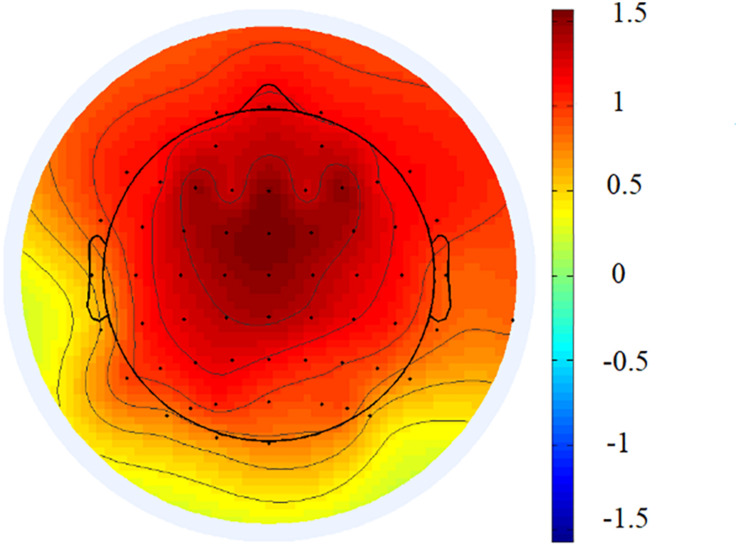
The grand average topographical distribution of theta/beta ratio.

Offline, the EEG data were re-referenced to the average mastoid reference and then filtered with low and high cutoffs of 0.01 and 100 Hz, respectively, and notch filtered between 49 and 51 Hz. Continuous EEG data were segmented into epochs of 1 s. Epochs contaminated by artifacts (eye blinks, eye movement) were corrected using independent component analysis. The independent components that accounted for artifacts were removed based on the scalp distribution (ocular activity projects mainly to frontal sites; Jung et al., 2000; Liu Y. et al., 2019). Epochs with a mean voltage exceeding ± 100 μV were removed as they included artifacts caused by eye blinks, muscle twitches, or body movements. The mean numbers and standard deviations of the epochs used for analysis were as follows: 107.98 ± 12.99 for the eyes-open condition; 107.65 ± 11.53 for the eyes-closed condition. The spectral power for resting-state EEG was obtained through a fast Fourier transform (epoch length 1 s, 50% overlap, Hanning window). FAA was then calculated as follows: ln (power_right_) − ln (power_left_). Given the inverse relation between the alpha power and activations, positive values would denote left activations, and negative values, right activations. Based on previous studies on the relation between emotion regulation and FAA (Wang et al., 2015; Choi et al., 2016; Papousek et al., 2017), we selected three frontal alpha asymmetry scores (FP2–FP1, F4–F3, and F8–F7)^2^. Based on the previous studies (Putman et al., 2010; Zhang et al., 2018) and the grand average topographical distribution (Figure 2), the slow/fast wave ratios were calculated by dividing the theta power density by the beta power density at the frontal electrodes (F3, FZ, and F4). Considering the non-normal distribution, power density values, and ratios were log normalized.

### Data Analysis

The NED for each participant was obtained by calculating the intra-class correlation^3^ (range = 0.43–0.97) between negative emotion ratings across the 20 images (Demiralp et al., 2012; Erbas et al., 2018; Nook et al., 2018; Kalokerinos et al., 2019). Fisher r-to-z transformations were then used to normalize these correlation coefficients. To facilitate understanding, we subtracted the resulting scores (range = −1.01 to 0.54; *M* = −0.004, *SD* = 0.39) from 1 so that large values would correspond to high NED and small ones to low NED.

In the data analysis, we employed descriptive statistics, Pearson’s correlation coefficient, and multiple regression methods. Mahalanobis distance values were used to determine the extreme values and no extreme values were found. Multicollinearity diagnostics between variables checked the variance inflation factor, eigenvalue, and tolerance values; no multicollinearity problem was found (all the variance inflation factors were below 5; all the tolerance values exceeded 0.1; most eigenvalues exceeded 0). We conducted multiple regressions to examine whether NED could predict the resting indicators of spontaneous emotional regulatory processes, with NED as the predictor, and FAA and TBR as dependent variables. The control variables (sex, age, handedness, anxiety, and depression) were first entered into the regression equation, followed by NED. Statistical analyses were conducted with SPSS 22.0 (IBM, New York, United States), with the significance level set at 0.05. To avoid type-1 errors, all the results applied the Bonferroni-corrected *p*-values.

We also conducted a control analysis to examine whether NED was specially related to alpha asymmetry and TBR in the frontal region. The same regression analyses for parietal alpha asymmetry (CP2–CP1, P4–P3, and P8–P7) and parietal TBR (P3, P4, and PZ) were conducted as in the frontal region to corroborate the findings that NED was linked to spontaneous emotion regulatory functioning, as indicated by resting EEG indicators.

## Results

### Frontal Alpha Asymmetry Results

The descriptive statistics and correlations between FAA and NED are presented in Table 1. The multiple regression analysis for FAA showed that NED significantly predicted the level of alpha asymmetry at prefrontal electrodes pairs (FP2–FP1; *t* = 2.64, *p_adj_* = 0.039, Table 2; F4–F3, *t* = 2.73, *p_adj_* = *0.03*, Table 2), with high-NED individuals showing a high level of alpha asymmetry. NED explained the significant variance of FAA at the FP2–FP1 (Δ*R*^2^ = 0.13) and F4–F3 (Δ*R*^2^ = 0.16) electrode pairs after accounting for variances due to control variables. The multiple regression analysis predicted alpha asymmetry in the frontal electrode pairs (F8–F7) which did not reach a significant level (*t* = 1.92, *p* = 0.064; Table 2).

**TABLE 1:** Bivariate correlations and descriptive statistics for FAA and NED.

Variables	1	2	3	4
1. NED	1			
2. FP2–FP1	0.31*	1		
3. F4–F3	0.35*	0.50**	1	
4. F8–F7	0.28*	0.49**	0.70**	1
*M*	−0.01	0.10	0.21	0.48
*SD*	0.39	0.15	0.18	0.27

NED, negative emotion differentiation. FP2–FP1 reflects the alpha asymmetry at FP2 minus that at the FP1 electrode site. F4–F3 reflects the alpha asymmetry at F4 minus that at the F3 electrode site. F8–F7 reflects the alpha asymmetry at F8 minus that at the F7 electrode site. *p < 0.05, **p < 0.01.

**TABLE 2:** Multiple regression analysis predicting FAA from NED.

Variables	β	*T*	*R*^2^	Δ *R*^2^	*F*
**FAA at PF2–PF1**					
Step 1: Control variable			0.27	0.27	2.34
Gender	0.16	0.97			
Age	0.28	1.79			
Handedness	−0.03	−0.19			
Depression	0.42	1.62			
Anxiety	0.04	0.16			
Step 2: Predictor			0.40	0.13	3.47*
NED	0.40	2.64*			
**FAA at F4–F3**					
Step 1: Control variable			0.17	0.17	1.30
Gender	0.19	1.06			
Age	0.18	1.06			
Handedness	0.15	0.85			
Depression	0.14	0.51			
Anxiety	0.14	0.50			
Step 2: Predictor			0.33	0.16	2.55*
NED	0.44	2.73*			
**FAA at F8–F7 pair**					
Step 1: Control variable			0.09	0.09	0.64
Gender	0.23	1.22			
Age	0.11	0.60			
Handedness	−0.02	−0.09			
Depression	−0.04	−0.15			
Anxiety	0.22	0.77			
Step 2: Predictor			0.19	0.10	1.20
NED	0.34	1.92			

FAA, frontal alpha asymmetry; NED, negative emotion differentiation. *p < 0.05. All the results applied the Bonferroni-corrected p-values.

Furthermore, to examine whether NED was specially related to alpha asymmetry in the frontal region, the same regression analysis for parietal alpha asymmetry (CP2–CP1, P4–P3, and P8–P7) was conducted. Results found that the indicators of parietal alpha asymmetry at CP2–CP1 (*t* = 1.83, *p* = 0.08), P4–P3 (*t* = 0.28, *p* = 0.78), and P8–P7 (*t* = −0.42, *p* = 0.68) were not predicted by NED.

### Theta/Beta Ratios Results

The correlation analyses between NED and frontal TBR and descriptive statistics are reported in Table 3. The multiple regression results found that NED significantly negatively predicted TBR at F3 (*t* = −2.60, *p_adj_* = 0.042; Table 4) and FZ (*t* = −2.53, *p*_adj_ = 0.05; Table 4) electrode sites, indicating that individuals with high NED presented a lower level of TBR. But the TBR at F4 could not be significantly predicted by NED (*t* = −2.12, *p_adj_* = 0.12; Table 4). Similarly, to examine whether NED was specially related to TBR in the frontal region, the control analysis for the TBR at the parietal electrode sites (P3, P4, and PZ) were conducted. Results found that NED could not predict the TBR at the P3 (*t* = −1.73, *p* = 0.09), P4 (*t* = −1.97, *p* = 0.06), or PZ (*t* = −1.81, *p* = 0.08) electrode sites.

**TABLE 3:** Bivariate correlations and descriptive statistics for the θ/β ratio and NED.

Variables	1	2	3	4
1. NED	1			
2. θ/β-F3	−0.40*	1		
3. θ/β-F4	−0.33*	0.95**	1	
4. θ/β-FZ	−0.36*	0.92**	0.94**	1
*M*	−0.01	1.31	1.31	1.55
*SD*	0.39	0.59	0.53	0.51

NED, negative emotion differentiation. θ/β-F3 reflects the θ/β ratio at the F3 electrode site. θ/β-F4 reflects θ/β ratio at the F4 electrode site. θ/β-FZ reflects the θ/β ratio at the FZ electrode site. *p < 0.05; **p < 0.01.

**TABLE 4:** Multiple regression analysis predicting θ/βfrom NED.

Variables	β	*T*	*R*^2^	Δ *R*^2^	*F*
**θ/β at the F3 electrode site**					
Step 1: Control variable			0.16	0.16	1.23
Gender	0.02	0.09			
Age	−0.15	−0.86			
Handedness	−0.04	−0.23			
Depression	−0.15	−0.53			
Anxiety	0.49	1.78			
Step 2: Predictor			0.31	0.15	2.34
NED	−0.43	−2.60*			
**θ/β at the F4 electrode site**					
Step 1: Control variable			0.22	0.22	1.82
Gender	0.10	0.55			
Age	−0.09	−0.57			
Handedness	−0.03	−0.20			
Depression	−0.04	−0.16			
Anxiety	0.50	1.90			
Step 2: Predictor			0.32	0.10	2.43*
NED	−0.34	−2.12			
**θ/β at the FZ electrode site**					
Step 1: Control variable			0.18	0.18	1.38
Gender	0.01	0.55			
Age	−0.12	−0.70			
Handedness	0.02	0.11			
Depression	−0.03	−0.11			
Anxiety	0.43	1.59			
Step 2: Predictor			0.32	0.14	2.40
NED	−0.41	−2.53*			

NED, negative emotion differentiation. *p < 0.05. All the results applied the Bonferroni-corrected p-values.

## Discussion

The results showed that NED significantly predicted the resting FAA and TBR of individuals. Individuals with high NED presented high FAA and low TBR. Spontaneous emotion regulation changes the intensity or duration of an emotional response in a natural, unintentional, and relatively effortless manner (Jackson et al., 2003; Egloff et al., 2006; Gyurak et al., 2011). Resting EEG data show spontaneous brain oscillation activities, and some indicators (e.g., FAA and TBR) reflect spontaneous emotional regulatory functioning (Jackson et al., 2003; Goodman et al., 2013; Tortellafeliu et al., 2014; Morillas-Romero et al., 2015). Thus, individuals with high NED were associated with enhanced spontaneous emotion regulatory functioning, validating the hypothesis of our study.

### Effect of Negative Emotion Differentiation on Frontal Alpha Asymmetry

Individuals with high NED showed high resting FAA at the prefrontal electrode pairs (FP2–FP1 and F4–F3), revealing that individuals with high NED presented more resting left-lateralized activations at these prefrontal regions. According to the three-stage neural network model of emotion regulation posed by Kohn et al. (2014), the dorsolateral prefrontal cortex plays a vital role in the initiation of emotion regulation. Some meta-analyses of functional magnetic resonance imaging studies have also indicated that the frontopolar is the vital activated brain region during cognitive reappraisal (Kalisch, 2009; Kohn et al., 2014). The FP2–FP1 and F4–F3 electrode pairs roughly lie at the frontopolar and dorsolateral prefrontal cortex, respectively. Thus, individuals with high NED presented left-asymmetrical activations at the prefrontal regions.

Increased resting left-lateralized activations at the prefrontal regions were associated with enhanced spontaneous emotion regulatory functioning. Individuals with high resting FAA at the electrode pairs (FP2–FP1) displayed heightened a spontaneous emotion regulation ability, as assessed by eye-blink startle magnitude after negative emotional stimulus offset (Jackson et al., 2003). Similarly, Goodman et al. (2013) found that individuals with high resting FAA at electrodes pairs (F4–F3) showed a high spontaneous emotion regulation ability under high-stress situations. A positron emission tomography study (Kim et al., 2012) revealed that greater left-sided bias in metabolic activity at the dorsolateral prefrontal cortex is associated with frequent use of a cognitive reappraisal strategy. Greater left prefrontal cortex activations likewise predict frequent use of cognitive reappraisal strategies (Wang et al., 2018). High FAA scores at the prefrontal electrode pairs (FP2–FP1) have been shown to predict the fluency and flexibility in generating cognitive reappraisals (Papousek et al., 2017). Thus, our finding indicated that individuals with higher NED were associated with enhanced spontaneous emotional regulatory functioning which is consistent with previous studies: individuals with high NED have an increased emotion regulation ability (Barrett et al., 2001; Kashdan et al., 2010; Erbas et al., 2018; Kalokerinos et al., 2019).

### Effect of Negative Emotion Differentiation on Theta/Beta Ratio

Consistent with our hypothesis, individuals with high NED showed low TBR. Low TBR is related to an increased attention control ability (Putman et al., 2010; Angelidis et al., 2016) and prefrontal cortex-mediated cognitive-emotional process (Keune et al., 2017; Angelidis et al., 2018; Van Son et al., 2018). The low TBR in individuals with high NED indicated their ability to suppress and control their own attention, which can promote spontaneous emotional regulation. In addition, low TBR is also indicative of the enhanced interaction between the frontal cortical and subcortical regions (Knyazev, 2007), which are the neural bases of the emotion regulation processes (Ochsner and Gross, 2007). Low TBR reflects heightened spontaneous emotional regulatory processes (Tortellafeliu et al., 2014). Thus, individuals with high NED presented low TBR, indicating their increased spontaneous emotional regulatory processes. Our findings were supported by previous studies which indicated that high NED decreased the intensity of negative emotion (Erbas et al., 2014; Lennarz et al., 2018; Kalokerinos et al., 2019) and maladaptive regulation strategies (Kashdan et al., 2010; Pond et al., 2012; Edwards and Wupperman, 2016) as spontaneous emotion regulatory functioning increased.

Based on previous studies (Jackson et al., 2003; Goodman et al., 2013; Tortellafeliu et al., 2014), the resting-state EEG indicators (FAA and TBR) reflected the ability of spontaneous emotion regulation, but these indicators were not equivalent to the spontaneous emotion regulation ability. Thus, we should be cautious when interpreting findings in this study. These findings only proved an association between NED and spontaneous emotion regulation.

### Significance

Taken together, these findings raise the possibility that the ability to differentiate discrete negative emotional states is linked to spontaneous emotion regulation indicative of the objective physiological indicators. Our findings provide the first resting-state neural evidence for the relation between NED and spontaneous emotion regulation which further corroborates this relationship from a physiological perspective. Additionally, our findings have important clinical implications. Our findings can be used to provide biomarkers for the emotion regulation intervention training of individuals with low NED. Increased resting FAA and diminished TBR can be regarded as biomarkers of improved spontaneous emotional regulatory functioning.

### Limitation and Future Directions

One limitation of this study is that only resting FAA was used. According to the capability model of frontal EEG asymmetry, state EEG asymmetry indicators (task-related FAA) may be more powerful than the trait ones in predicting individual differences, such as in terms of depression (Stewart et al., 2014) and affective style (Coan et al., 2006). Future research should combine trait and state FAA to examine the emotional regulatory function of NED. In addition, this study used the method of multiple regression to explore the relationship between NED and spontaneous emotion regulation indicative of resting-state EEG, with NED as a continuous variable. As this is a small-sample study, the variability range of NED scores was not very wide. To avoid this limitation, future study could increase the sample size and select low and high NED participants. Selecting participants could obtain the causal relationship between NED and spontaneous emotion regulation. Also, we are calling for future research that manipulates NED in the experimental condition, as in Cameron et al. (2013), and employs emotion regulation-related EEG indicators to determine the causal relationship between NED and emotion regulation. Lastly, only NED was investigated. Tugade et al. (2004) found that individuals with high positive ED showed greater psychological resilience when faced with stress. In addition, high positive ED could reduce emotion-related impulsivity and daily urges for maladaptive behaviors among individuals with borderline personalities (Dixon-Gordon et al., 2014). As such, individual differences in positive ED may also be linked with emotional regulatory functioning. Future studies could further explore this valuable issue.

## Conclusion

Individual differences in NED can predict resting spontaneous emotional regulatory functioning (FAA and TBR). Individuals with high NED presented high FAA and low TBR. High NED may be associated with enhanced spontaneous emotion regulatory functioning.

## Data Availability Statement

The raw data supporting the conclusions of this article will be made available by the authors, without undue reservation.

## Ethics Statement

The studies involving human participants were reviewed and approved by the Ethics Committee of Shanghai Normal University. The patients/participants provided their written informed consent to participate in this study.

## Author Contributions

YW and CS finished the data collection and preliminary analysis. YW, CG, and BH wrote the manuscript. All authors contributed to the article and approved the submitted version.

## Conflict of Interest

The authors declare that the research was conducted in the absence of any commercial or financial relationships that could be construed as a potential conflict of interest.

## Footnotes

1 The ID numbers of the IAPS pictures used were the following: 1301, 2751, 2399, 6510, 2100, 2130, 2810, 9320, 2278, 2276, 2272, 2715, 8466, 2722, 2301, 2410, 8231, 9429, 9075, and 2272.

2 By a mixed RM-ANOVA as the supplementary analysis with electrode location as a categorical within-subject variable and negative emotion differentiation (NED) Z-scores entered as a continuous between-subjects covariate, we did not find the interactive effects between electrode location and NED. Thus, electrode location was not regarded as a factor in the data analysis.

3 After the photo emotion differentiation task, each participant created a 20 × 20 matrix of the pictures and words. The intra-class correlation was computed based on the 20 × 20 matrix with absolute agreement between the negative emotion words across the 20 emotional pictures for each participant.
